# Online training for hearing implant surgery: A new approach to otological training

**DOI:** 10.1007/s00106-024-01452-9

**Published:** 2024-05-02

**Authors:** Kristen Rak, Stefan Kaulitz, Johannes Voelker, Franz-Tassilo Müller-Graff, Jonas Engert, Björn Spahn, Stephan Hackenberg, Peter Grasso, Rudolf Hagen

**Affiliations:** 1 Klinik und Poliklinik für Hals‑, Nasen- und Ohrenkrankheiten, plastische und aesthetische Operationen, Universitaetsklinikum Wuerzburg, Josef-Schneider-Straße 11, 97080 Wuerzburg, Germany; 2 MED-EL Elektromedizinische Geraete Gesellschaft m.b.H., Innsbruck, Austria

**Keywords:** Otologic surgical procedures, Online system, Temporal bone, Questionnaire, Simulation training, Otologische Eingriffe, Online-System, Felsenbein, Fragebogen, Simulationstraining

## Abstract

**Objective:**

Education in microsurgery of the ear includes staged training to allow for mastering of the complex microsurgical procedures, particularly in the context of middle ear reconstruction and cochlear implantation. Traditional surgical training includes temporal bone preparations by cadaver dissection and supervised operating room practice. As these on-site trainings are limited, there is a need to broaden education facilities in an on-line format. Therefore, a first basic on-line training for otosurgery was developed.

**Materials and methods:**

The system consists of an artificial temporal bone model together with a set of basic surgical instruments and implant dummies. As an essential part of the training kit, a high-resolution camera set is included that allows for connection to a video streaming platform and enables remote supervision of the trainees’ surgical steps by experienced otological surgeons. In addition, a pre-learning platform covering temporal bone anatomy and instrumentation and pre-recorded lectures and instructional videos has been developed to allow trainees to review and reinforce their understanding before hands-on practice.

**Results:**

Over the three courses held to date, 28 participants with varying levels of prior surgical experience took part in this otological surgical training program. The immediate feedback of the participants was evaluated by means of a questionnaire. On this basis, the high value of the program became apparent and specific areas could by identified where further refinements could lead to an even more robust training experience.

**Conclusion:**

The presented program of an otosurgical online training allows for basal education in practical exercises on a remote system. In this way, trainees who have no direct access to on-site instruction facilities in ear surgery now have the chance to start their otosurgical training in an educational setting adapted to modern technologies.

## Abstract (de)

**Ziel:**

Angehende Otologen stehen vor der Herausforderung, die komplexen mikrochirurgischen Techniken insbesondere im Zusammenhang mit Mittelohreingriffen und Cochleaimplantationen zu beherrschen. Traditionelle mikrochirurgische Ausbildungsmethoden beinhalten klassischerweise Präparationsübungen an Post-mortem-Präparaten und die begleitete Operation unter direkter Anleitung und Aufsicht. Dies setzt entsprechend voraus, Zugang zu Präparaten zu haben, und die Möglichkeit, von erfahrenen Chirurgen im eigenen Haus angeleitet zu werden. Da dies nicht ubiquitär umzusetzen ist, wurde ein Online-Trainingssystem für otologische Eingriffe entwickelt.

**Material und Methoden:**

Das System besteht aus einem künstlichen Modell des Schläfenbeins und den regulären chirurgischen Instrumenten und Übungsimplantaten. Zentraler Bestandteil ist eine hochauflösende Kamera, die mit einer Video-Streaming-Plattform verbunden ist und eine Online-Supervision der chirurgischen Schritte der Auszubildenden durch erfahrene Otochirurgen ermöglicht. Darüber hinaus wurde eine Pre-Learning-Plattform entwickelt, die die Anatomie des Felsenbeins, Information zum Instrumentarium sowie aufgezeichnete Vorlesungen und Lehrvideos umfasst, die es den Auszubildenden ermöglicht, ihr Wissen vor der praktischen Übung zu überprüfen und zu vertiefen.

**Ergebnisse:**

In den 3 bisher durchgeführten Kursen haben insgesamt 28 Teilnehmer mit unterschiedlicher chirurgischer Erfahrung an diesem Online-Fortbildungsprogramm für Otochirurgie teilgenommen. Mithilfe eines Fragebogens wurde die subjektive Bewertung des Programms durch die Teilnehmer abgefragt. So konnten auch Bereiche identifiziert werden, in denen eine Optimierung zu einer noch solideren Fortbildungserfahrung führen könnte.

**Schlussfolgerung:**

Das vorgestellte Programm einer otochirurgischen Online-Ausbildung kann dazu beitragen, bestimmte Basisgrundsätze im „Remote-Verfahren“ zu vermitteln. Damit wird diese Möglichkeit eines otochirurgischen Trainings auch Ärztinnen und Ärzten ermöglicht, die keinen unmittelbaren Zugang zu den klassischen Vor-Ort-Trainingseinheiten haben.

Surgeons and prospective otologists face the challenge of mastering the intricate and delicate procedures of otological surgery. Classic surgical training methods include temporal bone dissection and supervised operating room practice. In view of the urgent need for a more comprehensive and accessible approach to otological training for even remote regions, an online training system for microsurgery of the ear was developed.

## Concept

Otology, the branch of medicine specializing in diagnosing and treating diseases and disorders of the ear, occupies an essential place in surgical medicine. Proficiency in this field requires a deep understanding of anatomy and extensive hands-on experience [12]. Traditional surgical training methods often involve temporal bone dissection and supervised operating room practice, both of which are resource-intensive and limited in accessibility [2, 3, 5, 10, 11]. In an era of rapid technological advancement, online platforms have proven to be an invaluable asset in the education of medical professionals and the training of surgeons [9].

In view of the urgent need for a more inclusive and accessible approach to otological surgical training, a novel initiative was started: developing an online training system for otological surgeons using a portable temporal bone lab connected to the Internet. This innovative platform is specifically designed for middle ear and cochlear implant surgery—critical aspects of otological surgery. However, it is essential to note that the system’s potential extends well beyond these areas, providing a blueprint for developing online surgical training for various other surgical procedures.

One feature of this approach is the use of an artificial temporal bone model [7, 13] along with a set of surgical instruments and implant dummies. This represents a significant departure from many existing virtual training systems that rely solely on simulated environments [1, 8, 14]. By providing access to authentic, tangible elements of otological surgery, this platform offers an unparalleled hands-on experience in current virtual training.

The method also includes real-time supervision by experienced otological surgeons. Tutors can remotely follow the trainees’ surgical steps using a high-resolution camera connected to a video streaming platform. This capability allows for immediate and personalized advice and guidance. This integration of mentorship into the online training experience is a powerful tool that bridges the gap between traditional face-to-face surgical training and the benefits of online learning.

This online approach to training has the potential to benefit a broader range of patients by raising standards of care across different surgical specialties, thereby contributing to the advancement of surgical practice on a global scale. It also ensures that the acquisition of surgical expertise is not limited by geographical constraints [12].

## Materials

- Audio–video communication
- Flexible exoscopic macro-camera
- Artificial temporal bone specimen
- Surgical instrument pack
- Pre-learning platform
- Pre-recorded lectures and instructional videos:
- Questionnaire

Standard common online communication software facilitates real-time communication between trainees and experienced otological surgeons. This software provides seamless audio–video interaction, ensuring trainees can receive guidance, ask questions, and discuss their surgical procedures remotely with expert mentors.

**Fig. 1:**
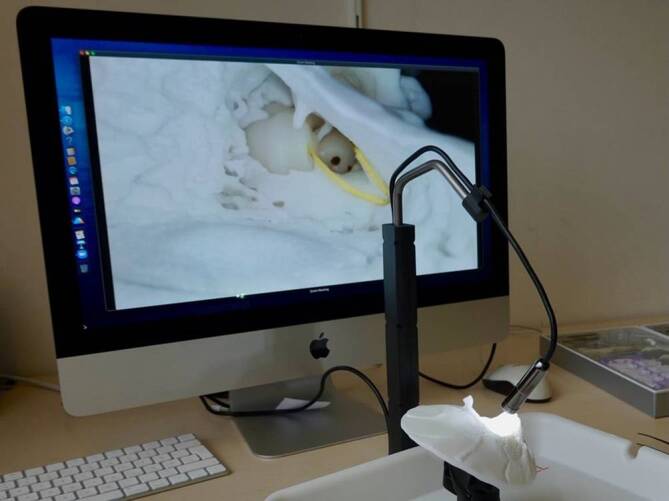
The Online Temporal Bone Lab consists of an audio–video communication system, a macro camera, and an artificial temporal bone specimen

A vital part of the setup is a high-resolution (5.0 megapixel, resolution 2594 × 1944 pixel) 2D macroscopic camera connected to a personal computer. This camera provides a detailed, close-up view of the surgical area, allowing trainees to closely observe and perform procedures with precision, which is critical to the accuracy and effectiveness of the training (Fig. 1).

**Fig. 2:**
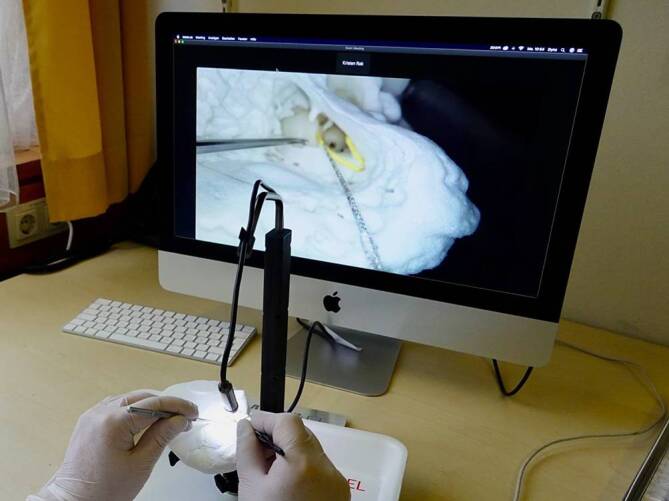
Online temporal bone training with on-screen visualization and online supervision

The artificial temporal bone specimen features an anatomically correct middle ear with ossicles and inner ear structures. To simulate the conditions of actual surgeries, the system includes an inner ear with a viscous fluid that mimics the fluids present in the cochlea. This feature allows trainees to practice electrode insertion through the round window in conditions resembling a live surgical environment (Fig. 2).

A package of critical surgical instruments is sent to trainees by post to ensure that trainees have all the necessary tools. These instruments have been carefully selected to replicate the tools used in otological surgery, providing trainees with a hands-on experience that closely mimics real-life procedures.

The online platform provides a comprehensive pre-learning program. This platform includes online lectures covering temporal bone anatomy and instrumentation. Trainees are provided with an explanation of how to set up the laboratory components to be well-prepared before starting their hands-on training.

To further enhance the learning experience, the pre-learning platform offers a repository of pre-recorded lectures and instructional videos. These resources illustrate each surgical step in detail, allowing trainees to review and reinforce their understanding before hands-on practice.

A questionnaire is administered to participants as an integral part of the training program. It enables the collection and evaluation of feedback, allowing for continuous improvement of the training program based on participant input. This structured questionnaire plays a vital role in improving the overall quality of the course and its relevance to the needs of otological surgeons and trainees.

The portable online training facility combines these elements to provide a holistic learning experience for otological surgeons. Trainees will be equipped with the essential materials, technology, and educational resources needed to become proficient in middle ear and cochlear implant procedures, while paving the way for the potential expansion of this approach to other surgical disciplines.

## Methodology

### Course schedule

In our approach to otological surgical training, we have adopted a blended learning model. Blended learning is a comprehensive educational approach combining different teaching methods to enhance the learning experience. Preparation materials are emailed to participants to ensure they have a clear understanding of the course content before it begins. A briefing session is organized to confirm that participants have received the necessary materials via the digital platform and by post, that their setup is correct, and that they are prepared for the course. The training (duration 3 h) begins with an introductory live session by the tutor explaining the course objectives, the skills to be acquired, and the importance of participant feedback. Throughout the course, surgical techniques are taught using a combination of video presentations and slides, with an emphasis on clarity and conciseness. Following each explanation, participants are divided into online groups, each with a dedicated tutor who provides real-time guidance and support to address questions and challenges as they arise. These breakout rooms group surgeons by language so that participants can receive support and engage in discussions in their native language whenever possible. This promotes a more comfortable and practical learning environment where participants can interact with peers with a common language and cultural context. The primary aim of this method is to build participants’ confidence by giving them practical, hands-on experience in handling real medical devices and working with anatomical models that closely simulate natural human anatomy.

### Evaluation

Following the exercises, the participants are provided with an online questionnaire via a QR code and asked to complete it. The evaluation is voluntary and anonymous, and thus there are no ethical concerns regarding the analysis of the data.

## Results

The training program has enjoyed considerable success and international participation. To date we have welcomed participants from several European countries, including Spain, Germany, and Austria. The diverse, global nature of these participants reflects the program’s ability to transcend geographical boundaries and provide access to training opportunities for otological surgeons from across the continent.

Over the three courses held to date, 28 participants have taken part in this otological surgical training program. Participants in these courses included individuals with varying levels of prior surgical experience, from those in the early stages of their otological training to those wishing to refine their skills. Their backgrounds spanned different medical institutions and practice settings, contributing to a rich and collaborative learning environment.

This diverse group of 28 participants demonstrated the program’s ability to attract a wide range of individuals with a common interest in advancing their knowledge and skills in otology. The success of the program lies in its ability to meet the needs of a broad and international audience, ultimately contributing to the growth and development of otological surgeons in different regions. The positive outcomes and international participation in these courses demonstrate the potential for further development and expansion of this innovative training approach.

**Fig. 3:**
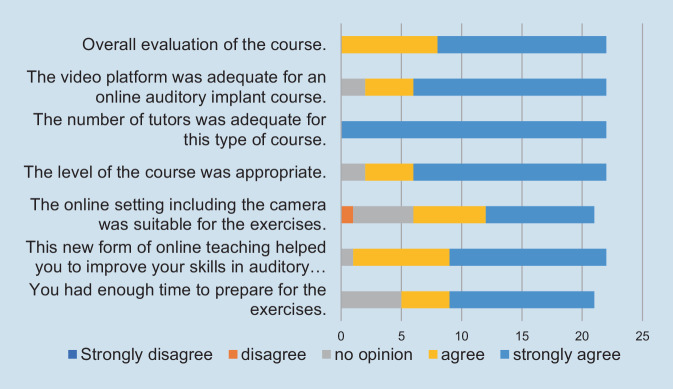
Evaluation of the questionnaire (total *n* = 24; missing values: unanswered questions)

The course was evaluated by means of a questionnaire. A total of 24 out of 28 participants answered the questionnaire. The mean overall evaluation of the course was 1.36 (1 *best*, 5 *worst*). For the other questions, the participants mostly strongly agreed or agreed with the course’s concept, design, and implementation (Fig. 3).

## Discussion

The positive feedback from participants in the otological surgical training courses underlines the value of the program. While the courses have been well received, there are specific areas where refinement can lead to an even more robust training experience.

One of the main challenges that arose during the training was the 2D view of the images seen through the macro camera. To overcome this challenge, moving to a 3D view with a head-up display would be a significant improvement. [6]. A 3D view would provide a more immersive and realistic perspective resembling the surgical environment. This transition would also greatly benefit the tutors and instructors guiding the students. The increased visibility in the 3D view would allow tutors to provide more precise and insightful guidance, facilitating a more thorough understanding of complex surgical techniques and intricate anatomical details.

Another aspect that can be improved is the temporal bone model used for training. The pre-drilled specimen used did not allow for haptic drilling training but simplified the logistics and avoided the generation of potentially harmful dust during drilling [4]. These barriers could be overcome by finding a way to simulate drilling without producing dust or by providing additional learning resources to familiarize participants with the drilling process. Several innovative ideas have been proposed to address this challenge and are currently being evaluated.

These enhancements have the potential to further improve the training program while addressing practical concerns associated with drilling in a nonclinical setting. As the field of otological surgery continues to evolve, adapting the training program to incorporate 3D views and improved bone drilling simulation will help participants develop even greater confidence and proficiency in their surgical skills.

The proposed solutions must be easy to implement and use so that the program remains accessible to a wide range of participants, including those with varying technical expertise.

## Practical conclusion

- The development of a portable online temporal bone lab connected to the Internet has provided unprecedented access to high-quality educational experiences for otologists, overcoming geographical limitations and enhancing the educational landscape.
- The use of blended learning techniques and personalized breakout rooms has promoted a dynamic and interactive learning environment, enabling participants from different backgrounds and countries to collaborate effectively. This approach has proven successful in building competence and confidence in the participants.
- However, participant feedback also highlighted the need for further improvements, particularly in replicating the tactile experience of drilling. Many creative ideas are being evaluated to meet this challenge, focusing on keeping the setup simple and feasible.
- By embracing cutting-edge technology and evolving methodologies, we can ensure that otological surgeons are well trained and well prepared to meet the evolving demands of the field, ultimately benefiting patients and the medical community.

## Declarations

For this article no studies with human participants or animals were performed by any of the authors. All studies mentioned were in accordance with the ethical standards indicated in each case.

The supplement containing this article is not sponsored by industry.
